# Modulation of Paracellular Permeability in SARS-CoV-2 Blood-to-Brain Transcytosis

**DOI:** 10.3390/v16050785

**Published:** 2024-05-15

**Authors:** Taylor E. Martinez, Karthick Mayilsamy, Shyam S. Mohapatra, Subhra Mohapatra

**Affiliations:** 1 Department of Molecular Medicine, Morsani College of Medicine, University of South Florida, Tampa, FL 33612, USA; tmartinez6@usf.edu (T.E.M.); karthick1@usf.edu (K.M.); 2 James A Haley VA Hospital, Tampa, FL 33612, USA; smohapat@usf.edu; 3 Department of Internal Medicine, Morsani College of Medicine, University of South Florida, Tampa, FL 33612, USA

**Keywords:** blood-brain barrier, SARS-CoV-2, transcytosis, permeability, ACE2, DPP4, neurovascular unit

## Abstract

SARS-CoV-2 primarily infects the lungs via the ACE2 receptor but also other organs including the kidneys, the gastrointestinal tract, the heart, and the skin. SARS-CoV-2 also infects the brain, but the hematogenous route of viral entry to the brain is still not fully characterized. Understanding how SARS-CoV-2 traverses the blood-brain barrier (BBB) as well as how it affects the molecular functions of the BBB are unclear. In this study, we investigated the roles of the receptors ACE2 and DPP4 in the SARS-CoV-2 infection of the discrete cellular components of a transwell BBB model comprising HUVECs, astrocytes, and pericytes. Our results demonstrate that direct infection on the BBB model does not modulate paracellular permeability. Also, our results show that SARS-CoV-2 utilizes clathrin and caveolin-mediated endocytosis to traverse the BBB, resulting in the direct infection of the brain side of the BBB model with a minimal endothelial infection. In conclusion, the BBB is susceptible to SARS-CoV-2 infection in multiple ways, including the direct infection of endothelium, astrocytes, and pericytes involving ACE2 and/or DPP4 and the blood-to-brain transcytosis, which is an event that does not require the presence of host receptors.

## 1. Introduction

SARS-CoV-2, the virus responsible for COVID-19, has provisional counts of over 110 million documented cases and 1.218 million deaths in the United States since January 2020 [https://www.cdc.gov/nchs/COVID19/index.htm (accessed on 28 February 2024)]. SARS-CoV-2 is an airborne respiratory virus that primarily infects the lungs via the ACE2 receptor but has well-documented secondary target organs including the kidneys, the gastrointestinal tract, the heart, the skin, and the brain [1]. While there is consensus that the brain is affected by the virus, the route of neuroinvasion remains controversial [2,3,4]. The two primary hypotheses are: (1) direct neuronal infection utilizing retrograde axonal transport via the cribiform plate and the olfactory bulb, and (2) transport into the brain across the blood-brain barrier (BBB) via active or passive transport mechanisms. While these two hypotheses are not mutually exclusive, the weight of evidence for the latter exceeds the former as the pandemic has progressed [3,4,5,6,7,8,9,10,11,12]. Understanding how SARS-CoV-2 interacts with the BBB is crucial to understanding the long-term effects that the pandemic will have on human health, as well as helping better prepare for future outbreaks of SARS-CoV-2 or different emergent coronaviruses.

The BBB is the cellular and molecular interface between the blood and the brain. It regulates the flow of nutrients, toxins, pathogens, and metabolic byproducts between the blood and the brain required for maintaining homeostasis [13]. At the cellular level, the BBB comprises (i) brain endothelial cells, which form the vascular lumen and are the primary interface for the BBB, (ii) pericytes, a stellate-like cell embedded in the basement membrane that wraps its processes around the endothelium and shares cytoplasm through gap junctions, and (iii) the end-feet of astrocytes which envelop 99.9% of the endothelium [14]. The BBB is characterized by several unique features including: highly impermeable intercellular tight junctions, highly active efflux pumps and an increased expression of transporters responsible for transporting homeostatically necessary nutrients at a seconds notice (e.g., GLUT1, ABC family transporters, amino acid transporters, etc.) [15,16,17,18]. Importantly, the brain endothelium phenotype is an induced phenotype that is mainly derived from signals received from pericytes and astrocytes [19,20,21,22]. This is an important feature of the BBB in the context of understanding SARS-CoV-2 infection since several groups have published on monocellular or bicellular models of the BBB [6,11,23], or iPSC-derived models [24,25,26]. Each of these lacks the intricate tri-directional signaling involved in BBB generation and maintenance or is artificially induced in the case of iPSC organoids, resulting in BBB models that are less representative of the in vivo phenotype, in addition to producing less translatable results.

Canonical SARS-CoV-2 infection occurs in the lungs through ACE2 anchoring and TMPRSS2-mediated cell membrane fusion initiated by cleavage between the S1/S2 peptides within the spike protein of the virus [27,28,29]. A logical extension of this knowledge is that SARS-CoV-2 will infect the BBB in the same way. However, clinical and postmortem studies remain inconsistent and inconclusive. Several studies have found ACE2 expression within the brain vasculature of glioblastomas [30], demonstrating a low total expression of ACE2 transcripts [31,32]. TMPRSS2 is further confounded with studies showing a wide variability in the expression of the transmembrane protease within the brain endothelium [32,33,34]. Lastly, some clinical postmortem studies have found that SARS-CoV-2 does make its way into the brain [12,33,35,36,37,38,39], yet the mechanistic avenue of hematogenous neuroinvasion remains elusive. Several groups have discovered that free spike protein is involved in disrupting tight junctions [40,41], and can be actively transported into the brain of mice [10], yet the understanding of intact viral interactions with the human primary cells of BBB is less well-characterized. 

*Coronaviridae* family infections in polarized cells have a feature that groups do not reconcile with some interpretations of the data—when coronaviruses infect polarized cells, they are secreted from the same side where the infection occurred (i.e., luminal infection results in the luminal release of new virions) [42,43,44,45,46,47]. The tension with this information is such that, if the endothelium of the BBB is directly infected, the virions will mature and exit into the lumen of the vasculature. Thus, understanding the requisite host features for the direct infection of cellular subtypes of the BBB as well as the differential cellular machinery producing transcellular movements remains incomplete. 

While it is known that ACE2 is involved in infection, many studies have shown that SARS-CoV-2 has features of receptor promiscuity that allow it to bind and infect using other receptors including neuropilin-1 [24,48,49], and alternate hypothesized receptors include Basigin [50], ASGR1, KREMEN1 [51], CD209 [52] and DPP4 (the primary receptor for MERS-CoV) [25,53]. This receptor promiscuity is also theorized as a means for how SARS-CoV-2 has been so successful in transmission as well as an explanatory feature for how the virus has been found in so many disparate tissues within postmortem studies [35,54]. It has been shown that astrocytes express DPP4, however, the group was unable to directly demonstrate DPP4’s role in direct infection [25].

The final component of SARS-CoV-2 infection is concerned with the protease(s) responsible for S1/S2 cleavage, thereby initiating virus-membrane fusion. Canonically, TMPRSS2 is the protease responsible for infection within the lungs [29,55,56] yet SARS-CoV-2 has been found in tissues that do not express TMPRSS2 leading to the discovery of secondary proteases involved in the S1/S2 cleavage within cells as opposed to the surface fusion TMPRSS2 is involved in. Alternative proteases include: furin [27,28,57] (secreted, as well as expressed within the trans-Golgi body) [57,58], CTSB, and CTSL (both constitutively expressed within the endosomal/lysosomal compartments of cells) [32,59,60]. Knowing these other proteases are expressed within organelles allows for two distinct methods of direct infection by SARS-CoV-2—cell surface membrane fusion and the release of viral RNA, or internalization, transport to the endosome/lysosome, and then viral-endosome fusion releasing viral RNA [61,62].

We hypothesize that the triumvirate of cells that comprise the BBB are both directly infected as well as involved in actively mediating the transcytosis of SARS-CoV-2 into the brain. We assessed the susceptibility of individual BBB cells (HUVEC, primary astrocytes, primary pericytes) to SARS-CoV-2 infection, as well as understanding their susceptibility in a transwell model of the BBB. Our results show the effects of SARS-CoV-2 infection on the BBB molecular mechanism involving paracellular permeability and transcytosis machinery that appears to function independently of direct infection. Collectively, these data suggest that the hematogenous neuroinvasion occurs independently of SARS-CoV-2 ACE2 anchoring.

## 2. Materials and Methods

### 2.1. Cell Culture

An HUVEC 10-donor pool was purchased from Lifeline CellTechnologies (FC-0044, Frederick, MD, USA) and primary astrocytes (#1800), and primary pericytes (#1200) were purchased from ScienCell (Carlsbad, CA, USA). Each cell type requires its own specialized media for cell growth. HUVECs were cultured in a VascuLife EnGS Endothelial Medium Complete Kit (LL-0002, Frederick, MD, USA), astrocytes were grown in specialized media Astrocyte Media [#1801], and pericytes were grown in specialized media Pericyte Media [#1201] both obtained from ScienCell (Carlsbad, CA, USA). VascuLife EnGS is supplemented with 2% fetal bovine serum and final concentrations of 30mg/mL gentamycin, 15 µg/mL Amphotericin B, 50 µg/mL Ascorbic Acid, 1 µg/mL Hydrocortisone hemisuccinate, 10 mM L-Glutamine, 5 ng/mL rhEGF, 0.75 U/mL Heparin Sulfate, and 0.2% EnGS LifeFactor (complete media abbreviated cEM). Astrocyte Media was supplemented with 2% FBS, 1% penicillin/streptomycin solution, and 1% proprietary astrocyte growth supplement (AGS, Cat. No. 1852, ScienCell, Frederick, MD, USA) and Pericyte Media was supplemented to final concentrations of 2% FBS, 1% penicillin/streptomycin, and 1% proprietary pericyte growth supplement (PGS, Cat. No. 1252, ScienCell, Frederick, MD, USA). The complete media is abbreviated as cAM and cPM, respectively. All cells were incubated at 37 °C in a humidified 5% CO_2_ incubator. 

Transparent PET, 0.4 µM pore Transwell inserts were obtained for 6-well (Cat. No. 83.3930.041), 12-well (Cat. No. 83.3931.041), and 24-well (Cat. No. 83.3932.041) plate applications from Sarstedt AG & Co. KG (Numbrecht, Germany). When using the transwell cell culture experimental design, HUVECs are seeded in the upper compartment of the transwell at enough cells to be 100% confluent the next day (1 × 10^6^ cells/well in 6 well, 4 × 10^5^ cells/well in 12 well, and 2 × 10^5^ in 24 well), and a 1:1 ratio of astrocytes to pericytes at 50% confluency are seeded into the lower compartment of the transwell plate. Prior to seeding HUVECs, transwell membranes were coated with 0.1 mg/mL Cultrex Poly-L-Lysine (Cat. No. 3438-100-01, BioTechne, Minneapolis, MN, USA), according to the manufacturer’s protocol. cEM was used in the upper compartment of the transwell. In the lower compartment, a 1:1 ratio of cAM to cPM was used to grow the 1:1 ratio of astrocytes to pericytes. 

### 2.2. SARS-CoV-2 Infections

For monolayer infections, cells were seeded at least 24 h prior to infection, and up to 96 h prior to infection for the shLentivirus experiments, to allow the knockdown to take effect. All infections were initiated at 1 multiplicity of infection (MOI) and were ended at 48 h post infection unless otherwise noted utilizing icSARS-CoV-2-mNG (SARS-CoV-2 stably encoding mNeonGreen) provided from Dr. PEI-Yong Shi from the University of Texas Medical Branch, Galveston, TX, USA [63]. IcSARS-CoV-2 was engineered by inserting a Neon Green reporter gene into ORF7. IcSARS-CoV-2-mNG exhibited no change in titer, plaque morphology, viral RNA profile, and replication kinetics compared to the wild-type virus it was generated from the 2019-nCoV/USA_WA1/2020 strain (GenBank: MT246667.1). For the transwell experiments, HUVECs were seeded at 95% confluency in the upper well and astrocytes and pericytes seeded at 30–40% in the lower well 5 days prior to infection to allow cells to reach confluency and for tight junctions to mature. Infections were conducted overnight, and the media were refreshed first thing the next morning. All experiments were conducted in a BSL-3 environment. 

### 2.3. Plaque Assay

Viral titers were quantified by determining the number of individual plaque-forming units (PFUs) after 72 h of infection on confluent Vero-E6 ACE2 TMPRSS2 cells. A total of 100 µL viral stock was added directly onto Vero-E6 ACE2 TMPRSS2 cells or diluted 1:1 in 100 µL OptiMEM and serially diluted to 1:5, 1:10, or 1:20 final dilutions in OptiMEM serum-free media. Next, viral-media dilutions were inoculated on 7.5 × 10^4^ Vero-E6 ACE2 TMPRSS2 cells in quadruplicate in a 48-well plate for 3 h. The inoculum was then removed and replaced with 0.8% carboxymethylcellulose in OptiMEM + 2% FBS + 1% penicillin-streptomycin antibiotic as overlay media for 72 h. The plates were then fixed in 80% methanol in water for 20 min, rinsed with 1× PBS, and stained with 0.1% crystal violet solution in ethanol. Plaques were then counted and calculated at PFU/mL. 

### 2.4. Quantitative Reverse Transcriptase PCR (qPCR)

Cells were collected in Trizol, and total RNA was isolated according to manufacturer’s protocol and subsequently quantified using the 2000 Nanodrop spectrophotometer (ThermoScientific, Waltham, MA, USA). The Verso cDNA synthesis kit (cat. No. AB1453A, ThermoScientific, Waltham, MA, USA) was used to synthesize cDNA. The PCR amplification for qPCR utilized Forget-Me-Not Eva Green qPCR master mix on a Bio-Rad CFX-384 thermocycler following the manufacturer’s instructions. The analysis of qPCR curves utilized the CFX Maestro software (Version 2.0) to calculate Δ_cq_ and ΔΔ_cq_, by normalizing gene expression to housekeeping genes, beta-actin or glyceraldehyde 3-phosphate dehydrogenase (GAPDH). PCR primer sequences are listed in Table 1.

### 2.5. Lentivirus Protocol

#### 2.5.1. Transfection

HEK-293T cells were utilized for plasmid transfection of shRNA plasmids containing shACE2 (TRCN0000046694, TRCN0000046696, TRCN0000046697) or shDPP4 (TRCN0000050773, TRCN0000050776, TRCN0000372047) sequences in a pLKO.1 (Cat. No. 8453, Addgene, Watertown, MA, USA) backbone co-transfected with the second generation lentiviral plasmids psPAX2 (Cat. No. 12260, Addgene, Watertown, MA, USA) and pMDG.2 (Cat. No. 12259, Addgene, Watertown, MA, USA). First, HEK293T cells were seeded 24 h prior to transfection on a 100mm cell culture dish. Next, plasmids were mixed in 500 µL optiMEM at a ratio of 6.4 µg of target plasmid: 6.4 µg of psPAX2: 3.2 µg pMDG.2 with 32 µL of P3000 reagent (Cat. No. 100022058, Invitrogen, Waltham, MA, USA). Since 3 shRNA plasmids were simultaneously used in each transfection, the 6.4 µg of target plasmid was divided by 3 parts equally among the 3 plasmids, i.e., 2.13 µg of each target plasmid. Then, 20 µL of Lipofectamine 3000 (Cat. No. 100022052, Invitrogen, Waltham, MA, USA) was added to 500 µL of OptiMEM and then the DNA-P3000 mixture was added to the Lipofectamine 3000 mixture, vortexed for 5 s, and incubated at room temperature for 20 min to ensure complexing. After incubation, the transfection mixture was added gently dropwise to the 100 mm dish containing 5.5 mL OptiMEM and incubated in the cell culture incubator overnight. The next morning, the transfection mix was aspirated and replaced with 6 mL complete growth medium (DMEM + 10% FBS + 1% anti/anti). 

#### 2.5.2. Harvest and Purification

After 8 h, the supernatant was harvested, the cells were replenished with 6 mL of fresh complete DMEM, and the collected supernatant was centrifuged for 10 min at 500× *g* to remove cell debris. Next, the clarified supernatant was moved to a fresh tube and placed in a bucket of ice; the bucket of ice was placed in the 4 °C fridge for the next clarified supernatant harvest. After 16–24 h, the supernatant was harvested again from the transfected HEK293T cells, and centrifugation and clarification steps were repeated. From this point forward, supernatant was maintained at an ice-cold temperature. Clarified supernatants were combined into a single tube and a Lenti-X Concentrator was added to the supernatant at a 4:1 ratio (12 µL of supernatant requires 4 µL of Lenti-X Concentrator). The tube was inverted several times until Lenti-X was fully mixed with the supernatant and placed on ice for at least 15 min. After the incubation was complete, the tube was centrifuged for 45 min at 1500× *g* and a small white pellet formed; this was the lentivirus. The supernatant was aspirated and the lentiviral pellet was resuspended in 1/10th the supernatant volume with sterile PBS. To ensure a better long-term storage of lentivirus, with minimal loss upon thawing, Lenti-Guard was added at a 1:4 ratio to the PBS (300 µL Lenti-Guard to 1200 µL PBS). Next, the lentivirus was aliquoted into single-use volumes and stored at −80 °C for long-term storage. 

#### 2.5.3. Lentiviral Titer and Transduction

During every lentiviral transfection, a concurrent GFP lentivirus was created as a proxy for determining lentiviral infectious units per mL (IU/mL). Briefly, the GFP lentivirus was harvested as previously described, and immediately placed into a 96-well plate of HEK293T cells seeded at 15,000 cells/well for transduction. Each row of the plate was serially diluted with the concentrated GFP lentivirus from 10^−1^ to 10^−6^ (10 µL lentivirus into 90 µL OptiMEM). The viral media was then aspirated 24 h later, and replaced with a fresh batch of GFP lentivirus, and 48 h post-transduction the cells were imaged for GFP fluorescence. The green cells are counted and compared to the amount of nonfluorescent cells and IU/mL is calculated using these numbers. Using this IU/mL, the shACE2 and shDPP4 lentivirus are used at 1 IU/mL for knockdown. 

For knockdown, the lentivirus is placed on cells for 24 h and replaced with a fresh batch, and analysis for knockdown is conducted at 72 h from the first exposure to lentivirus, as this time point marks the beginning of SARS-CoV-2 infection if the experimental design includes a knockdown condition. 

### 2.6. Dextran Permeability Assay

To assess the paracellular permeability and the effectiveness of tight junctions in the transwell-BBB model, we used various sized fluorescent Dextran molecules. First, the two dextrans, Texas Red 3000 MW (D3329, Invitrogen, Waltham, MA, USA) and Cascade Blue 10,000 MW (D1976, Invitrogen, Waltham, MA, USA) were diluted in sterile, molecular grade H_2_O to a concentration of 1 mM for 3 kDa, and 10 mM for 10 kDa. From the stock solutions, the dextrans were diluted in cEM to their working concentrations of 1 µM and 10 µM, respectively. From there, the dextran-spiked media were placed onto the HUVECs in the upper compartment. To measure the dextran pass through, media from the lower compartment is sampled at 1/100th of the total volume at t_0_ = 0 h, t_1_ = 24 h, and t_2_ = 48 h. The media is then collected into a black 96-well plate in triplicate and their respective excitation–emission spectra are read. The readings are averaged and normalized within the bounds of 100% (dextran-spiked cEM) permeability in an empty well, and 0% permeability (t_0_). Using the permeability coefficient calculation, all coefficients are calculated using area, concentration, and time of sampling using the equation below where *P_app_* is the permeability coefficient, *V_basolateral_* is the volume of media in the lower well in cm^3^, Δ*C_basolateral_* is the change in concentration (μM) of dextran in the lower well, Δ*t* is the time in seconds, *A* is the surface area of the transwell insert, and *C_apical_* is the concentration of the dextran-spiked media at t_0_.

(1) $$P_{app}=\frac{V_{basolateral}\;\left(\frac{\Delta C_{basolateral}}{\Delta t}\right)}{A\times C_{apical}}$$

### 2.7. Immunocytochemistry

Cells were fixed with freshly prepared 4% PFA for 30 min, then washed twice with PBS. If necessary, cells were permeabilized in 2% BSA, 0.1% TritonX-100, and in PBS for 10 min at room temperature, and then blocked for 1 h at room temperature using 2% BSA. Cells were washed twice with PBS for 2 min each cycle, and then incubated with the desired primary antibody (Table 2) in 2% BSA in PBS overnight at 4 °C on a side-to-side shaker. Once removed from 4 °C, the primary antibody was removed, and cells were washed twice with PBS for 5 min. Next, the appropriate secondary was diluted 1:1000 in PBS and incubated at room temperature for 1 h on a shaker in darkness. The cells were finally counterstained using DAPI diluted 1:2000 in PBS for 15 min and then washed 3 times in PBS before being taken for imaging. 

### 2.8. Western Blots

Cells were collected using RIPA buffer (Cat. No. 89901, ThermoScientific, Waltham, MA, USA) supplemented with HALT protease and phosphatase inhibitor cocktail (78440, ThermoScientific, Waltham, MA, USA) and a cell scraper. Cell lysis mixtures were then sonicated using a Branson digital sonifier at 26% amplitude for 6 s three times per sample. Next, debris was centrifuged at 16,000× *g* for 30 min at 4 °C, and the supernatant was moved to new tubes for protein concentration quantification. Total protein quantification was completed using a Pierce Coomassie protein assay kit (Cat. No. 23200, ThermoScientific, Waltham, MA, USA) according to the manufacturer’s instructions. Then, 10 to 30 µg of total protein or molecular weight standards were resolved using SDS-polyacrylamide gel electrophoresis and transferred to a 0.45 µm nitrocellulose membrane (Cat. No. 1215458, GVS North America, Sanford, ME, USA) at 100 V for 2 h. The blot was then blocked in 5% nonfat milk in TBS-T for 1 h at room temperature. Each blot was then incubated with a solution of primary antibody (Table 2) at the proper dilution overnight at 4 °C on a side-to-side rocker. After washing the blot three times with TBS-T for 5 min each, the blot was then incubated with the appropriate secondary HRP antibody in 5% nonfat milk in TBS-T for 2 h at room temperature. After the secondary antibody incubation, blots were washed three times again in TBS-T for 5 min. Protein bands were visualized using SuperSignal West Pico PLUS chemiluminescent substrate (Cat. No. 34580, ThermoScientific, Waltham, MA, USA) and imaged using the ChemiDoc XRS imaging system according to the manufacturer’s instructions. 

## 3. Results

### 3.1. HUVECs, Primary Human Astrocytes, and Primary Human Pericytes Are Susceptible to SARS-CoV-2 Infection

**Figure 1:**
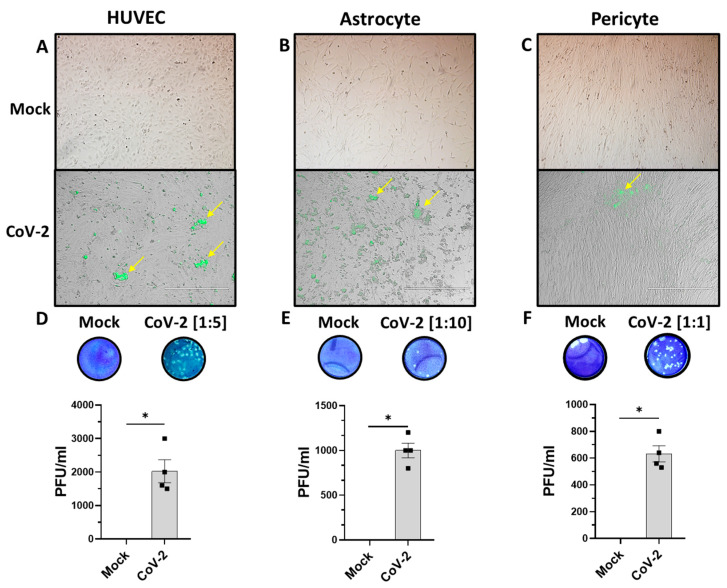
Cellular components of BBB are productively infected with icSARS-CoV-2-nMG. (**A**–**C**) Live-cell imaging of HUVECs, astrocytes, and pericytes mock infected or infected with 1 MOI of icSARS-CoV-2-nMG at 24 hours post infection (hpi). Brightfield and green fluorescent images are merged to demonstrate infection within cells. Yellow arrows indicate infected cells. (**D**–**F**) Representative plaque assay images. Numbers indicate dilution of cell culture supernatant from virus-infected cells. Mean plaque assays conducted with 2 replicates from the supernatant of infected samples at 48 hpi. Scale bars = 400 μm. * *p* < 0.05 as measured using Student’s *t*-test.

First, we aimed to determine if the BBB cells were susceptible to SARS-CoV-2 infection. We exposed a monolayer of each cell type (HUVECs, and primary astrocyte and pericyte) to 1 MOI of icSARS-CoV-2-nMG and monitored the infection via fluorescent live cell imaging and measured productive infection through plaque assays using the supernatant from the mock and infected cells on VERO-TMPRSS2-ACE2 cells. Fluorescent live cell imaging showed a noticeable infection at 24 h, with minor to moderate cell pathology in the overlaid brightfield images (Figure 1A–C). We showed that SARS-CoV-2 produces a small but productive infection in HUVECs (PFU_HUVEC_ = 2025 PFU/mL) and smaller still in astrocytes and pericytes (PFU_astrocyte_ = 1000 PFU/mL, PFU_Pericyte_ = 632.5 PFU/mL) (Figure 1D–F).

**Figure 2:**
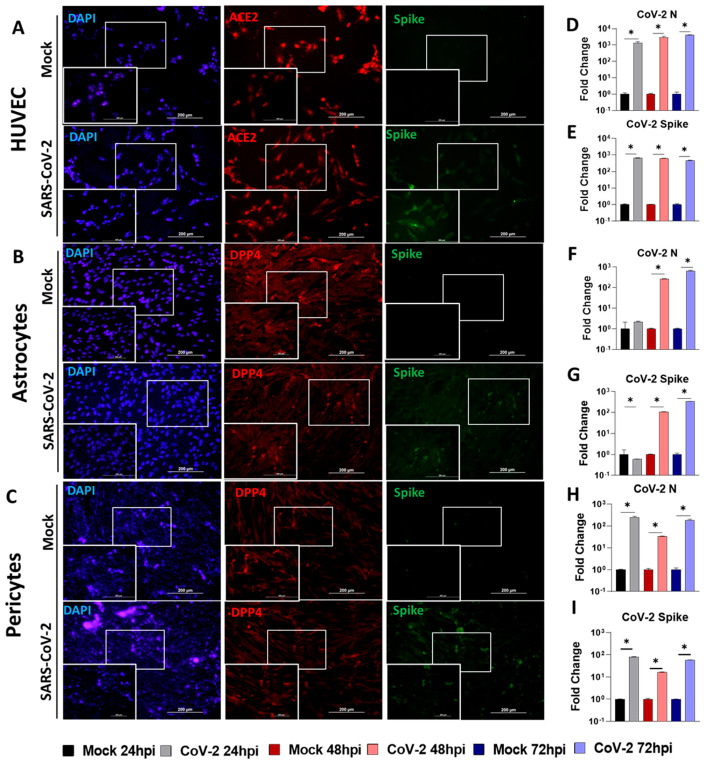
HUVECs, astrocytes, and pericytes were infected with 1 MOI of icSARS-CoV-2-nMG. Cells were fixed 48 hpi and immunostained. Cell pellets harvested at 24, 48 and 72 hpi used for qPCR analysis. (**A**) Immunostaining of infected HUVECs with ACE2 and SARS-CoV-2 spike antibodies. (**B**,**C**) Immunostaining of infected astrocytes and pericytes with DPP4 and SARS-CoV-2 spike antibodies. (**D**–**I**) qPCR analysis of N and spike mRNA as a measure of infection at 24, 48, and 72 hpi. * *p* < 0.05 as measured using One-Way ANOVA and subsequent post hoc Fisher’s LSD test.

To examine which host receptor and protease are involved in SARS-CoV-2 infection of the target cell, we infected HUVECs, and primary astrocytes, and pericytes for up to 72 h and collected cells for qPCR analysis. For imaging, we fixed the cells at 48 hpi and immunostained with antibodies to ACE2, DPP4 or spike protein. We found that HUVECs expressed ACE2 (Figure 2A) but not DPP4 (Figure S1) and ACE2 expression did not change after SARS-CoV-2 infection. Further, spike protein expression was seen upon SARS-CoV-2 infection (Figure 2A). Moreover, the qPCR data showed that SARS-CoV-2 rapidly infects HUVECs (Figure 2D,E), and mRNA for both N and spike were detected within 24 hpi and were maintained at least until 72 hpi; furthermore, they were significantly different (*p* < 0.05) compared to their mock counterparts in all three measured time points. We did not see any expression of ACE2 in astrocytes and pericytes (Figure S1), however, these cells expressed DPP4 (Figure 2B,C). Also, N and spike RNA were detected in both astrocytes and pericytes (Figure 2D–I); however, the timeline of detection in astrocytes began at 48 hpi. In pericytes, there was a significant difference (*p* < 0.05) at 24 hpi. Collectively, the data demonstrate the SARS-CoV-2 susceptibility of each cell type.

### 3.2. Lentiviral Knockdown of ACE2 in HUVECs and DPP4 in Astrocytes and Pericytes with Minimal Off-Target Effects

Using a lentiviral protocol, we developed three lentiviruses that can knockdown either ACE2 or DPP4 via encapsulating shACE2 or shDPP4 plasmids in HUVECs, and primary astrocytes and pericytes. The corresponding lentivirus encapsulating scrambled sequences were used to develop shScr plasmids that were used as controls. We transduced each cell type with an equivalent of 1 IU/cell for 48 h and assessed the effects of knockdown and potential off-target effects on proteases involved in SARS-CoV-2 infection (Figure S1). HUVECs expressed ACE2, TMPRSS2, CTSB, and furin, but not DPP4. Further, the transduction of HUVECs with shACE2 lentivirus reduced ACE2, with no other effects on the other proteins measured. Astrocytes did not express ACE2, and transduction of these cells with shDPP4 lentivirus reduced the expression of DPP4 (Figure S1). We also noticed an effect on furin expression, while TMPRSS2 and CTSB exhibited no modulation via lentiviral transduction. Pericytes also did not express ACE2, and we found DPP4 knockdown upon exposure to shDPP4 lentivirus (Figure S1). We also found a slight downregulation effect on furin similar to what we observed in astrocytes. 

### 3.3. Lentiviral Knockdown of ACE2 in HUVECs and DPP4 in Astrocytes and Pericytes Ablates SARS-CoV-2 Infection

To determine the effects of ACE2 depletion in HUVECs or DPP4 depletion in astrocytes and pericytes on infection with SARS-CoV-2, we first transduced the shLentiviruses on their respective cells for 48 h at the 1 IU/cell and then infected cells with 1 MOI SARS-CoV-2. Culture supernatants were used for plaque assays and cells were harvested for qPCR or Western blot analysis. 

**Figure 3:**
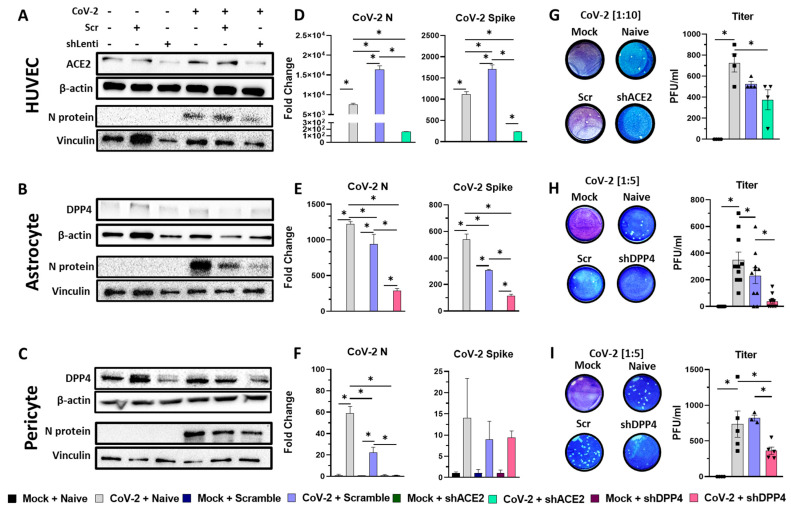
Knocking down ACE2 in HUVECs and DPP4 in astrocytes and pericytes reduces infection burden. Cells were transduced with shACE2- or shDPP4-lentiviruses or corresponding shScr lentiviruses (1 U/cell) for 48 h prior to infection with SARS-CoV-2 (1 MOI). (**A**–**C**) Western blots of ACE2, DPP4 and N protein expression in HUVECs astrocytes and pericytes. (**D**–**F**) qPCR measurements of viral mRNA for N and spike mRNAs of SARS-CoV-2 infected cells compared with their mock counterparts. (**G**–**I**) Representative plaques from each experimental condition for each cell type, and their quantifications in PFU/mL. * *p* < 0.05 using One-Way ANOVA and subsequent post hoc analysis with Fisher’s LSD Test.

The confirmation of ACE2 depletion in HUVECs (Figure 3A) and DPP4 depletion in astrocytes and pericytes (Figure 3B,C) in shACE2 or shDPP4 transduced cells were confirmed using Western blotting. The treatment with shScr lentivirus in each of these cells did not alter the expression of ACE2 or DPP4. Further, SARS-CoV-2 infection did not increase the expression of ACE2 or DPP4 in these cells. QPCR data suggested that knocking down ACE2 in HUVECs and DPP4 in astrocytes and pericytes resulted in reductions in mRNAs for N and spike proteins compared to naïve cells as well as the shScr lentivirus treated cultures (Figure 3D–F), suggesting decreased productive replication. 

This was further confirmed using plaque assays (Figure 3G–I). The analysis of the plaque assay in SARS-CoV-2 infected HUVECs (Figure 3G) suggested that there was a significant reduction in plaques from the naïve vs. shACE2 conditions (*p* < 0.05). However, there was a nonsignificant difference between the naïve and shScr lentivirus transduced HUVECs, as well as the scramble and shACE2 transduced cultures (*p* = 0.0506). In SARS-CoV-2 infected astrocytes (Figure 3H), pre-transduction with shDPP4 lentivirus significantly reduced virion production (34 PFU/mL) vs. shScr lentivirus (231 PFU/mL) vs. naïve astrocytes (348 PFU/mL), demonstrating a pronounced effect of DPP4 in permitting a productive infection from SARS-CoV-2. Similarly, we found a pronounced reduction in SARS-CoV-2 infection in pericytes pre-transduced with shDPP4 lentivirus (Figure 3I) (362 PFU/mL) vs. shScr lentivirus (820 PFU/mL) vs. naïve pericytes (736 PFU/mL).

### 3.4. SARS-CoV-2 Productively Infects the Luminal and Abluminal Compartments of a Transwell BBB Model without Altering Paracellular Permeability

We then wanted to determine the dynamics at play in a transwell BBB model with HUVECs in the upper compartment and astrocytes and pericytes co-cultured in the lower compartment. We exposed the upper compartment to a 1 MOI SARS-CoV-2 infection for 48 h and measured the infection with qPCR, evaluated the paracellular permeability with 3 kDa and 10 kDa fluorescent dextran molecules, and lastly quantified the plaques from each compartment to determine the level of productive infection. 

**Figure 4:**
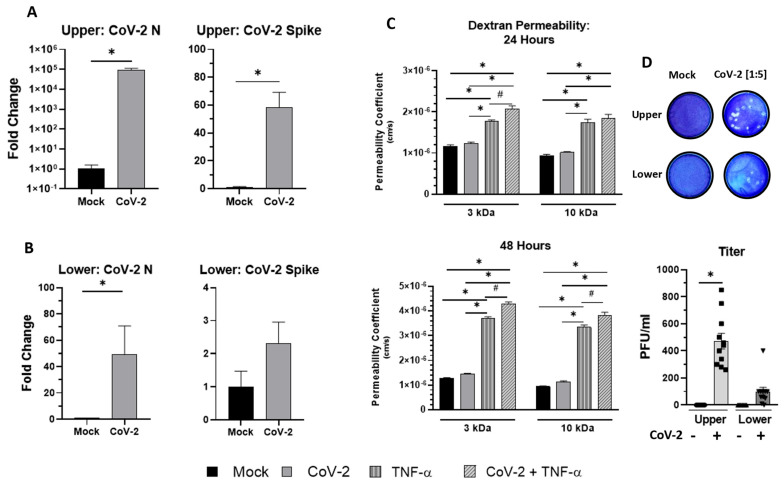
Transwell BBB model is infected in both compartments by SARS-CoV-2 while maintaining paracellular integrity. (**A**,**B**) qPCR analysis of mRNA of N and spike in the upper (HUVECs) and lower wells (astrocytes and pericytes) of the model. (**C**) The permeability coefficient of passive diffusion of 3 kDa and 10 kDa fluorescent dextran molecules placed in the upper compartment of the BBB model to the lower compartment 24 hpi and 48 hpi. The raw reads of fluorescence were converted to a permeability coefficient using Equation (1) mentioned above. (**D**) Representative images of plaque assays and their corresponding dilution factor. Supernatants from the upper and lower compartments of the transwell were sampled at 48 hpi and measured in a plaque assay with infectious viral titer being reported in PFU/mL. #, * *p* < 0.05 using Student’s *t*-test or One-Way ANOVA and subsequent post hoc analysis with Fisher’s LSD Test.

We found that the infection significantly (*p* < 0.05) increased the detection of SARS-CoV-2 N and spike RNA 48 hpi in the transwell upper compartment (Figure 4A). In the lower well of the transwell, we detected N and spike transcripts, however, only the N expression was significantly increased (Figure 4B). 

After confirming a productive infection in the transwell BBB model, we wanted to determine if the direct infection of the upper well alters the paracellular permeability. TNF-α (10 ng/mL) was used as a positive control in this experiment. We found that SARS-CoV-2 infection in the upper well did not alter the paracellular permeability of either dextran-sized molecule (3 kDa, 10 kDa) at either time measured despite our expectations (Figure 4C). 

Lastly, we examined plaque assays with the supernatant from the upper and lower compartments of the transwell BBB model (Figure 4D). We found a significant (*p* < 0.05) number of plaques derived from the upper compartment (470 PFU/mL) but an insignificant (*p* = 0.1093) number of plaques in the lower well (98.18 PFU/mL). While statistically insignificant, it did indicate that the virus passes through the HUVEC monolayer in the luminal well without the disruption of the paracellular tight junctions, suggesting that active transport may be responsible for virions in the abluminal side. 

### 3.5. SARS-CoV-2 Transcytosis across the BBB Is a Process Mediated through Clathrin and Caveolin Endosomes

To determine how viral particles move across the BBB without the disruption of paracellular permeability, we used inhibitors of the endocytosis and endosomal transport process (Table 3) in conjunction with an ACE2 lentiviral knockdown to isolate the effects that ACE2 and/or endocytosis play in SARS-CoV-2 transcytosis. We have selected (i) PNGase F that catalyzes the cleavage of N-linked oligosaccharides between the innermost GlcNAc and asparagine residues of high mannose, hybrid and complex oligosaccharides from N-linked glycoproteins, (ii) chlorpromazine (CPZ) that inhibits clathrin-mediated endocytosis, (iii) filipin that inhibits caveolin-mediated endocytosis, (iv) brefeldin A (BFA) that disrupts the trans-Golgi body, and (v) monensin that inhibits the function of the late endosome/lysosome by preventing acidification. Collectively, these drugs will allow us to determine the type of endocytosis, avenues of intracellular transport, and the roles organelles play in transcytosis.

**Figure 5:**
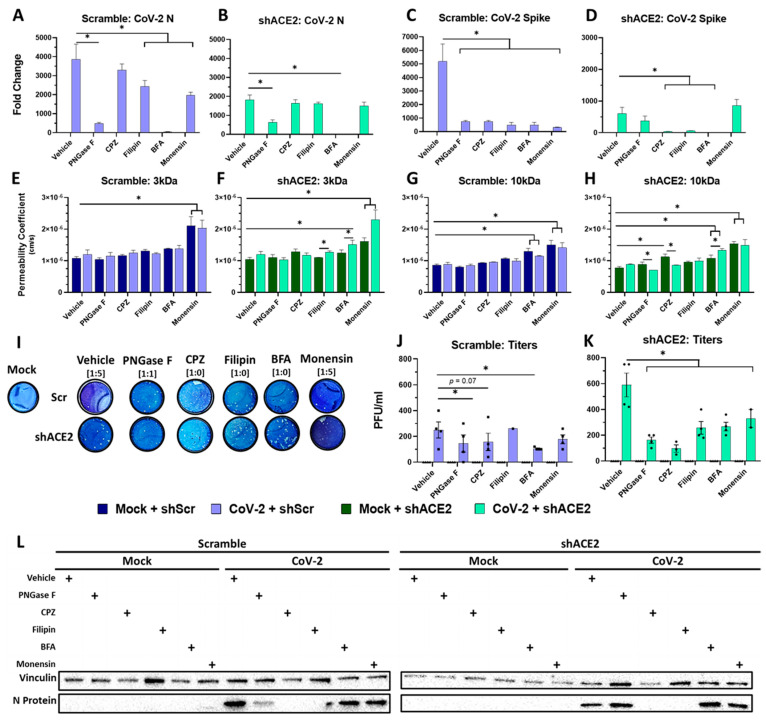
SARS-CoV-2 transcytosis across the BBB is a process mediated through clathrin and caveolin endosomes irrespective of ACE2 expression. (**A**–**D**) qPCR measurements of infection in the upper well quantifying N and spike mRNA expression 16 hpi demonstrating varying levels of infection in both shScr + CoV-2 and shACE2 + CoV-2. Drug conditions are compared with vehicle group. (**E**–**H**) Differences (or lack thereof) in paracellular permeability measured using a 3 kDa and 10 kDa fluorescent dextran molecule placed in the upper compartment of the BBB model and passive diffusion of the molecules to the lower compartment. The raw reads of fluorescence were converted to permeability coefficients using Equation (1) mentioned above. (**I**–**K**) Representative images of plaque assays and their corresponding dilution factor. Supernatant from the lower compartment of the transwell was sampled at 16 hpi and measured in a plaque assay with infectious viral titer being reported in PFU/mL. (**L**) Detection of N protein in the cells (astrocytes and pericytes) of the lower well using Western blots. All mock conditions demonstrate no detection, while varying levels of N were seen within the CoV-2 conditions. Detection of N protein in lower well cells was not wholly correlated with plaque assays but broadly aligns. * *p* < 0.05 using Two-Way ANOVA and subsequent post hoc analysis with Fisher’s LSD Test.

First, we incubated HUVECs in the upper compartment with shACE2- or shScr-lentivirus for 48 h, and then, prior to infection, we incubated cells with PNGase F at 1 U per 300 cells for 4 h, CPZ at 25 µM for 2 h, filipin 2 µg/mL for 2 h, BFA at 1 µg/mL for 2 h, or monensin at 5 µM for 2 h. Utilizing crystal violet, we verified monolayer health and attachment after exposure to their respective lentiviral conditions, pharmacologics, and infection (Figure S2). We exposed the transwell BBB model to 1 MOI SARS-CoV-2 for 16 h to observe any transcytosis phenomenon before the first full replication cycle of SARS-CoV-2, which is approximately 22 h [23,64,65,66]. As a verification that SARS-CoV-2 had not been fully replicated yet, we analyzed subgenomic RNA for N and spike which is a marker for actively replicating virus (Figure S3). We found that subgenomic N transcripts were detected, but subgenomic spike transcripts were not, indicating that a full replication cycle had not been completed yet. In the absence of any inhibitors, the transduction of shScr lentivirus (CoV-2 + Scramble) showed a significant increase in N and spike in the upper wells compared to mock; however, the exposure of shACE2 lentivirus (CoV-2 + shACE2) showed a significant reduction in both N and the spike transcripts compared to the shScr control (CoV-2 + scramble) (Figure 5A–D). The corresponding conditions measured using plaque assay from the lower wells showed the inverse effect—the transduction of shACE2 lentivirus significantly increased plaques in the lower well compared with its scramble counterpart (Figure 5I–K). 

Next, we examined the effects of inhibitors of endocytosis and endosomal transport in BBB transcytosis. Looking at infection in the upper well in the presence of PNGase F (Figure 5A–D), we found a significant reduction in N and spike transcripts in the shScr and shACE2 conditions. Additionally, PNGase F (Figure 5I,K) reduced the viral titer in the lower well in the shScr and shACE2 conditions compared to the corresponding vehicle conditions (Figure 5J,K). 

When analyzing the effects of CPZ in infection in the upper well, we found that in the presence of CPZ, the N transcript was not different from the vehicle-treated groups in the shScr and shACE2 conditions (Figure 5A,B). In contrast, we found a significant reduction in spike mRNA in shScr and shACE2 conditions (Figure 5C,D). Measuring the viral titer in the CPZ-treated group showed no difference in the shScr viral titers (Figure 5I,J), but a significant reduction in viral titer in the shACE2 condition (Figure 5I,K); the inclusion of CPZ disrupted the increased transcytosis seen in the vehicle + shACE2 condition.

The addition of filipin to the transwell BBB model had similar effects to CPZ. We found a significant reduction in the N and spike transcripts in CoV-2 + shScr condition compared to the vehicle (Figure 5A,C). The fold change in N transcripts in the CoV-2 + shACE2 condition was no different than the vehicle condition (Figure 5B). However, we did find a reduction in spike transcripts in the CoV-2 + shACE2 group (Figure 5D). Looking at viral titers in the filipin-treated group, we found that there was no effect on viral titers in the CoV-2 + shScr group compared to vehicle condition (Figure 5I,J). However, we found a significant reduction in viral titer in CoV-2 + shACE2 group (Figure 5I,K).

Next, in the upper well, we found that BFA-induced Golgi body disruption across all infection groups resulted in a significant reduction in N and spike transcripts compared to the vehicle control (Figure 5A–D). We also found that BFA disrupted tight junctions in 3kDa measurements of CoV-2 + shACE2 (Figure 5F), and in 10 kDa measurements in all four experimental groups assayed (Figure 5G,H). Interestingly, we also found significant reductions in viral titers in both CoV-2 + shScr and CoV-2 + shACE2 groups (Figure 5I–K), despite the disrupted paracellular permeability, suggesting a role of the trans-Golgi body on both infection in the upper well and transcytosis to the lower well. 

Lastly, the function of the lysosome in direct infection and transcytosis is not fully clear. In the presence of monensin, we found a diminished N and spike mRNA expression in the CoV-2 + shScr condition (Figure 5A,C), but that reduction was not seen in CoV-2 + shACE2 (Figure 5B,D). Looking at the titers in the presence of monensin, we found that there was no difference in CoV-2 + shScr compared with the vehicle (Figure 5I,J). However, we did find a significant reduction in the CoV-2 + shACE2 group compared to the vehicle group (Figure 5I,K). In most of the other drug + lentivirus conditions, we found the permeability coefficient stay at or around 1 × 10^−6^ cm/s (Figure 5E–H), indicating no paracellular tight junction disruption allowing us to make observations about direct infection and transcytosis in tandem, but in the presence of monensin we did see an increase in permeability which prevents us from making similar observations (Figure 5E–H).

A summary of the detection of the nucleocapsid protein in the cells of the lower compartment (Figure 5L) showed the diminished detection of N protein in infected cells pre-exposed to PNGase F, CPZ, and filipin. Upon shACE2 lentiviral transduction, we found the effect of PNGase F was canceled out by ACE2 knockdown, but the CPZ and filipin effects were retained. 

## 4. Discussion

In this study, we report several novel findings important to understanding how SARS-CoV-2 interacts with the BBB and how the virus undergoes transcytosis to the abluminal side of the brain. First, we confirmed that infection is possible in endothelial cells (HUVEC) via the canonical infection process (ACE2-TMPRSS2), which was reported during the initial onset of the pandemic in January 2020 by Hoffman et al. [29]. Second, we determined that the most likely primary receptor for infection within astrocytes and pericytes is DPP4, which is also the receptor for MERS-CoV. Third, knocking down ACE2 in HUVECs, and DPP4 in astrocytes and pericytes diminishes but does not completely ablate infection. Fourth, transcytosis occurs regardless of ACE2 expression via glycocalyx anchoring and clathrin and/or caveolin-dependent manner.

Here, we successfully depleted DPP4 and suggest a direct link of DPP4-mediated SARS-CoV-2 infection of astrocytes and pericytes which agrees with other reports [67,68]. Also, we found that the transduction of shDPP4 lentivirus exhibited a reduction of furin expression (Figure S1). While some work has demonstrated furin’s role in priming, this effect seems to be variable and cell type dependent, and the knockdown or inhibition of furin may be overcome by other proteases in receptor-mediated infection processes [27,69]. However, since shDPP4 resulted in a partial knockdown, it will be prudent to include DPP4 competitive binding assays or direct DPP4 antibody interference in the presence of SARS-CoV-2 to elucidate a direct link more clearly. There also remains a debate about the role of DPP4 in receptor-mediated infection. It appears that DPP4 plays a small but noticeable role in BBB infection (and only in the absence of ACE2) since gliptin drugs have been demonstrated to directly interfere with the binding site exploited by the SARS-CoV-2 spike [67]. 

There is still some controversy about how SARS-CoV-2 produces the neurological symptoms associated with acute and chronic forms of the disease. Our results affirm that these symptoms can be linked with a direct infection of the endothelium and the brain-side pericytes and astrocytes from the virus whereas other groups suggest that symptoms are downstream of an inflammatory response from the rest of the body [3,9,70]. While such inflammatory symptoms and direct infection are not mutually exclusive, our evidence suggests that acute and chronic brain disease states can be independent of the systemic inflammatory response and associated cytokine storm, particularly derived from the initial lung infection. 

The primary cells of the BBB used in our model are prone to de-differentiation after removal from their BBB niche [71,72,73,74,75]. This rapid de-differentiation following isolation plays a role in protein expression profiles. The results of our monolayer studies exhibit slightly different protein expression profiles than cells examined within the BBB niche. Also, we selected HUVECs instead of brain endothelial cells in our BBB experiments due to their ability to be induced by the BBB-like niche created when tri-cultured with astrocytes and pericytes [76,77,78,79,80].

While transwell models represent an adequate model for BBB experimental designs, they are categorized as having lower biological relevance than in vivo [81] or custom-designed microfluidic BBB devices [74,82]. However, our BBB model did not include several physiological features, including the physical association of pericytes [83] with endothelial cells, and shear stress [84]. Further validation of our findings requires experiments in such more complex BBB models. 

In many cases of viral infection of the endothelium of the BBB, there is a corresponding tight junction disruption resulting in BBB paracellular “leakage” [85,86,87,88]. Several groups early in the pandemic using different dosages of spike protein found that there is a significant decrease in the measured tightness of tight junctions based on transendothelial electrical resistance (TEER) as well as the expression of overall proteins involved in tight junction architecture (Claudin-5, ZO-1) [9,40,41,89]. However, other groups used a whole intact virus, they found that the paracellular tight junctions remained intact [23,90], which is consistent with our findings regarding paracellular permeability. Thus, tight junction disruption appears to be related to disease model type (spike vs. intact virions), cell type (primary vs. cell line), as well as SARS-CoV-2 variant dependence such as the WT, alpha, beta, delta, omicron isolates. This leads us to speculate that the c-terminus of the S1 peptide may be the subviral component responsible for tight junction disruption. While normally bound to S2 and hidden from interaction with cells [91], when dosed in isolation, it appears to have the largest effects on tight junction disruptions. Extending this logic, the shedding of S1 peptide during acute infection may also be an important factor in the disparate findings in laboratory results as well as BBB permeability status in clinical settings [92,93]. 

Furthermore, the finding that ACE2 depletion increases transcytosis with a corresponding decrease in infection in the luminal side of the transwell suggests a likelihood of an inverse relationship between ACE2 expression and levels of transcytosis. However, this requires further validation. This finding places ACE2 expression on the luminal side of the BBB in a central role for understanding how acute and chronic infection of the brain parenchyma may occur. Additionally, N-acetylglucosamine (GlcNAc) residues seem to play a supporting role in multiple processes regarding SARS-CoV-2, including direct infection and transcytosis. As with many viruses, the glycocalyx and proteoglycan shell of cells typically provide a ‘sticky’ molecular environment for viruses to anchor to before interacting with their specific receptors. Several studies describe a key role of GlcNAc in transcytosis, likely playing an anchoring role via ionic interactions i.e., a negatively charged SARS-CoV-2 binds to the positively charged glycocalyx [80,81]. However, the understanding of how genetic manipulation or post-translational effects [94] play a role in SARS-CoV-2 infection and transcytosis remains mechanistically unclear. Future avenues of research should try to categorize the roles of the glycosylation of ACE2 and the general cellular glycosylation status to decipher how SARS-CoV-2 interacts with the surface of the BBB. Since pharmacological inhibition is a less targeted method of controlling cellular functions due to off-target effects, our results suggest that the genetic disruption of processes may assist in further categorizing the types of transcytosis involved at the BBB. 

In addition, our findings suggest that infection with SARS-CoV-2 increases the transcellular routes of the blood-to-brain movement of SARS-CoV-2 and therefore may be partially responsible for brain infection. One study used a mouse model for SARS-CoV-2 infection and showed that Cav-1 plays a role in the increased unidirectional movement of macromolecules, and this increased transcellular phenotype was rescued with a Cav-1 knockout mouse [95]. This increased expression of Cav-1 was also discovered in our analysis of postmortem COVID-19 patients, further strengthening the role caveolin-mediated transcytosis could be playing in SARS-CoV-2 infection [96]. These findings are in agreement with other reports attempting to understand how SARS-CoV-2 moves across the BBB-injected mice with S1 protein, and described adsorptive transcytosis [10,97]—a process often mediated by caveolin—as the mechanism underlying the movement. 

Moreover, clathrin has been implicated to be important in both infection and transcytosis [98]. A study utilizing spike protein and pseudotyped SARS-CoV-2 virus demonstrated that clathrin is sufficient for internalization, although a caveolin-mediated avenue was not ruled out [61]. Importantly, clathrin and caveolin are both involved in many different types of internalization, transportation, and transcytosis processes within cells and delineating the location of each endosome requires further experimentation. Due to the accumulation of evidence for both caveolin and clathrin in these processes, it warrants much more study into how the components of SARS-CoV-2 (spike, nucleocapsid, envelope, and their variants) may be deterministic in cell localization and/or transcytosis. 

Overall, in our model, luminal BBB expression of ACE2 appears to operate as a molecular sponge, which can sequester a circulating virus and increase the proportion of luminal infection to and luminal viral secretion, which can then ultimately be managed and neutralized by the systemic immune response and counterintuitively play a role in protecting from SARS-CoV-2 transcytosis into the brain. The protective effect of ACE2 expression and overexpression is well documented in the *Coronaviridae* family of viruses [77], as well as the corresponding depletion leading to worse outcomes in both peripheral and CNS tissues in SARS-CoV-1 and SARS-CoV-2 infection [12,78]. 

## 5. Conclusions

**Figure 6:**
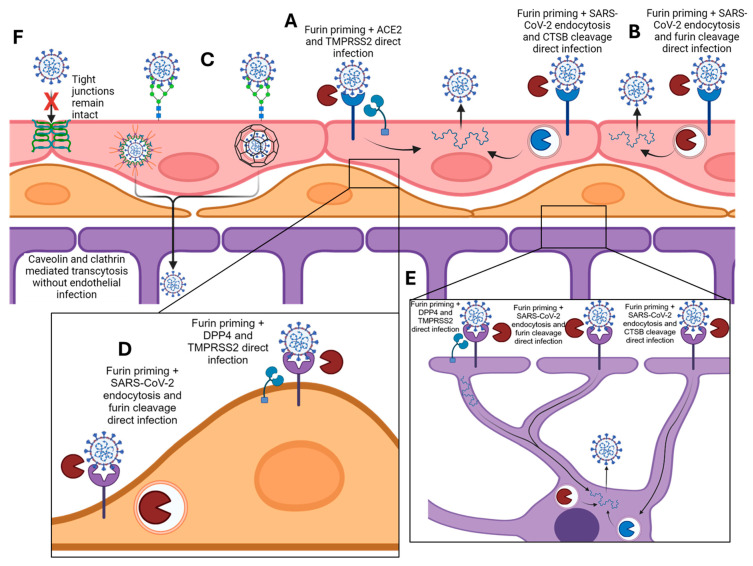
Proposed model of SARS-CoV-2 infection and transcytosis of the BBB. (**A**,**B**) Direct infection of brain endothelial cells including ACE2 binding, furin priming, and S1/S2 cleavage by either TMPRSS2, or CTSB and/or furin. (**C**) Transcytosis with initial anchoring to glycocalyx, endocytosis by either caveolin or clathrin, and deposition on abluminal side of endothelium. (**D**,**E**) Proposed mode of infection of pericytes and astrocytes utilizing DPP4 binding, furin priming, and TMPRSS2 spike cleavage, or DPP4 binding and endocytosis for spike cleavage within endosome. (**F**) Diagram showing intact tight junctions preventing passive diffusion of SARS-CoV-2.

Summarizing our results, SARS-CoV-2 progresses through a decision tree when interacting with the BBB. First, if ACE2 and TMPRSS2 are present at the luminal surface of the BBB, it can result in a direct infection of the endothelium. If ACE2 is present without TMPRSS2, then the BBB internalizes the virion, anchored by ACE2 binding, and produces a direct infection through the secondary targets of the endosome—likely the Golgi body. Lastly, if ACE2 is not present, but the virion becomes anchored through a separate molecular interaction, likely the glycocalyx, the probability of transcytosis increases and is mediated through either clathrin or caveolin. In sum, we propose a model for BBB-SARS-CoV-2 interactions including the direct infection of the cellular components of the BBB (Figure 6A,B,D,E), the transcytosis of SARS-CoV-2 through the BBB via glycocalyx anchoring and clathrin and/or caveolin endosomes (Figure 6C), and a lack of paracellular permeability increase (Figure 6F).

Thus, the BBB is susceptible to SARS-CoV-2 infection in multiple ways. The direct infection of endothelium, astrocytes, and pericytes, the three discrete cell types that comprise the BBB, is possible through ACE2 and DPP4, respectively. The direct infection of the endothelium does not appear to modulate the paracellular permeability of the BBB. Finally, SARS-CoV-2 can undergo transcytosis even with a diminished expression of ACE2 on the luminal surface of the endothelium, leading to a deposition of virus in the brain parenchyma via caveolin and clathrin-mediated endocytosis.

## Figures and Tables

**Table 1:** Sequences of primers used in qPCR.

Gene Name	Species	Sequence
*N*; *ORF9a*	Virus	5′-CAC ATT GGC ACC CGC AAT C-3′ 5′-GAG GAA CGA GAA GAGGCT TG-3′
*S*	Virus	5′-CTT CCC TCA GTC AGC ACC TC-3′ 5′-AAC CAG TGT GTG CCA TTT GA-3′
*ACE2*	Human	5′-GGA CCC AGG AAA TGT TCA GA-3′ 5′-GGC TGC AGA AAG TGA CAT GA-3′
*DPP4*	Human	5′-GCA CGG CAA CAC ATT GAA-3′ 5′-TGA GGT TCT GAA GGC CTA AAT C-3′
β-*ACTIN*	Human	5′-CAC CAT TGG CAA TGA GCG GTT C-3′ 5′-AGG TCT TTG CGG ATG TCC ACG T-3′
*GAPDH*	Human	5′-CCA TGT TCG TCA TGG GTG T-3′ 5′-CCA GGG GTG CTA AGC AGT T-3′
*Subgenomic N*	Virus	TRS-B sequence 5′-ACA AAC CAA CCA ACT TTC GA-3′
		5′-GAA TCT GAG GGT CCA CCA AA-3′
*Subgenomic Spike*	Virus	TRS-B sequence 5′-ACA AAC CAA CCA ACT TTC GA-3′ 5′-GCA GGG GGT AAT TGA GTT CT-3′

**Table 2:** Antibodies used in experiments.

Antibody	Cat. #	Antibody	Cat. #	Antibody	Cat. #
SARS-CoV-2 Nucleocapsid, Rabbit Monoclonal ab	40143-R004	Anti-DPP4 [11D7]	Ab114033	Goat anti-Rabbit IgG (H+L), FITC	65-611
Anti-SARS-CoV-2 S2, Goat IgG	AF10774	Anti-Cathepsin B [EPR4323]	Ab125067	AlexaFluor 596 Donkey anti-Mouse (H+L)	A21203
Rabbit polyclonal Ab to ACE2	Ab15348	Anti-TMPRSS2 [EPR3862]	Ab109131	Goat anti-Rabbit IgG (H+L) Super Clonal, AlexaFluor 647 conjugate	A27040
Beta-Actin (C4) mouse IgG	Sc-47778	Anti-Furin antibody	Ab3467	AlexaFluor 647 donkey anti-rabbit IgG (H+L)	A31573
Vinculin (H-10) Mouse monoclonal IgG	Sc-25336	Anti-Cathepsin L+V [33/2]	Ab6314	AlexaFluor 488 donkey anti-Mouse IgG (H+L)	A21202
Goat polyclonal Ab to DPP4	Ab62816	Goat anti-Mouse IgG/IgM HRP	AP130P	SARS-CoV/SARS-CoV-2 Nucleocapsid Antibody, Mouse monoclonal Ab	40143-MM05
Goat anti-Rabbit IgG (H+L) HRP	AP307P	AlexaFluor 488 Donkey anti-Goat IgG (H+L)	A11055		

**Table 3:** Drugs used in transwell BBB transcytosis experiments and their molecular functions.

Drug Name	Cat. #	Function
PNGase F, Recombinant	P0708S; New England Biolabs (Ipswich, MA, USA)	Enzymatic deglycosylation; cleaves between the innermost N-Acetylglucosamine (GlcNAc) and asparagine residues of high mannose, hybrid, and complex oligosaccharides from N-linked glycoproteins.
Chlorpromazine (hydrochloride)	16129; Cayman Chemical (Ann Arbor, MI, USA)	Prevents the assembly and disassembly of clathrin lattices on cell surfaces and endosomes.
Filipin	25073; Cayman Chemical (Ann Arbor, MI, USA)	Binds to cholesterol, a component of caveolae, and disrupts caveolar formation.
Brefeldin A	11861; Cayman Chemical (Ann Arbor, MI, USA)	Causes fragmentation of the Golgi apparatus.
Monensin (sodium salt)	16488; Cayman Chemical (Ann Arbor, MI, USA)	A Na+/H+-exchanging ionophore that prevents acidification of lysosomal-like organelle compartments.

## Data Availability

All data generated or analyzed during this study are included in this published article (and its Supplementary Materials).

## Supplementary Materials

The following supporting information can be downloaded at: https://www.mdpi.com/article/10.3390/v16050785/s1, Figure S1: Western blots of receptors and proteases involved in SARS-CoV-2 infection and expression after shLentivirus; Figure S2: Crystal violet staining of transwells used in lentivirus and drug experiments demonstrating intact monolayers; Figure S3: Raw cycle of amplification values for genomic and subgenomic RNA from upper wells in lentivirus and drug experiments.
