# Supplementary material for: Modulation of Paracellular Permeability in SARS-CoV-2 Blood-to-Brain Transcytosis
Source: Viruses. 2024 May 15;16(5):785. doi: 10.3390/v16050785 (PMC11126142; doi:10.3390/v16050785)
Supplement: Supplementary file 1 [file viruses-16-00785-s001.zip › viruses-2959471-supplementary.pdf]

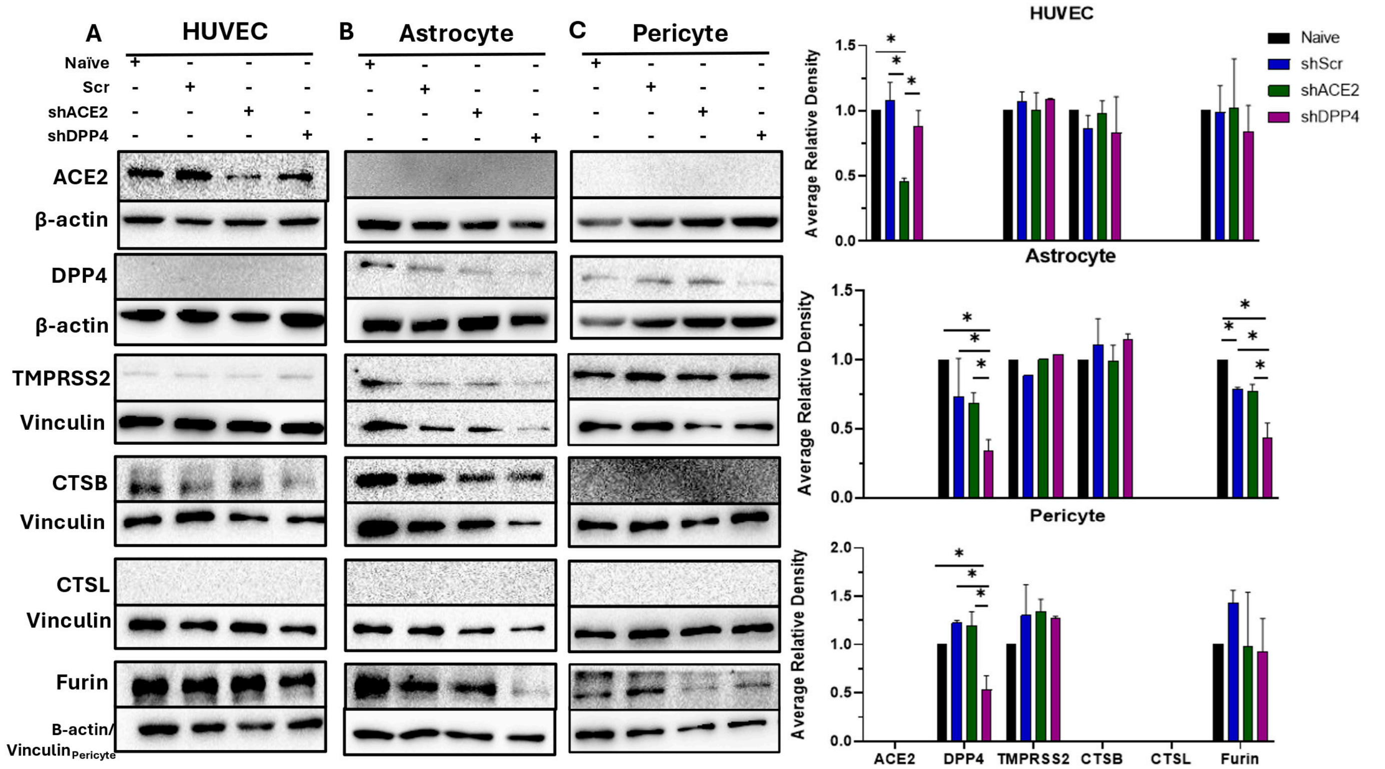

Supplementary Figure 1. Western Blots of receptors and proteases involved in SARS-CoV-2 infection and expression after shLentivirus. HUVECs, astrocytes and pericytes were exposed to scrambled- (Scr), shACE2-, or shDPP4-lentivirus for 48 hrs and cell pellet examined by western blotting for indicated proteins. Each target protein is aligned with a corresponding loading control of β-actin (45kDa), or vinculin (124 kDa). Densiometric analysis of the bands presented as well. * $p < 0.05$, 2way ANOVA, post hoc Fisher's LSD test.

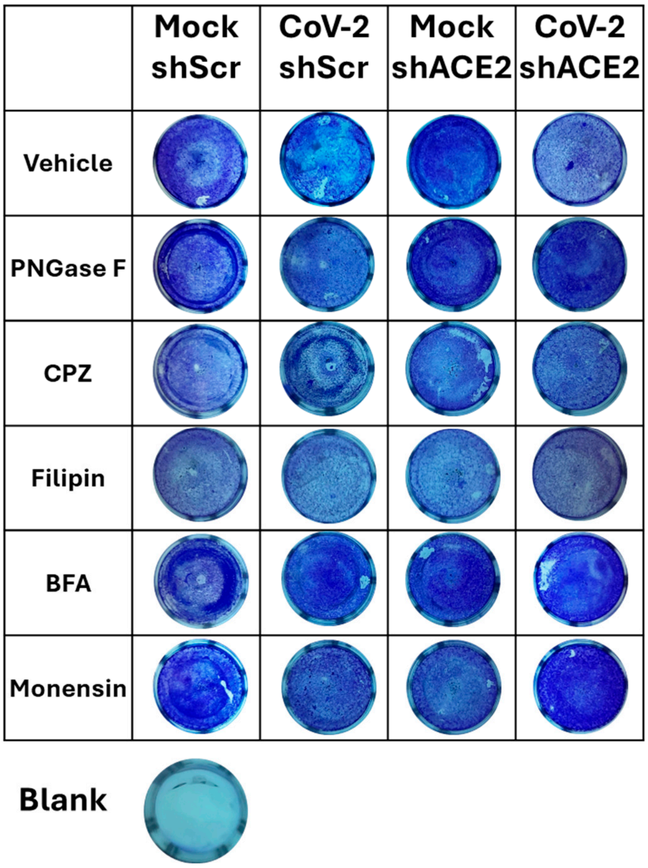

Supplementary Figure 2. Crystal Violet staining of transwells used in lentivirus and drug experiments demonstrating intact monolayers in upper well. Transwells were fixed and stained after 16-hour SARS-COV-2 infection.

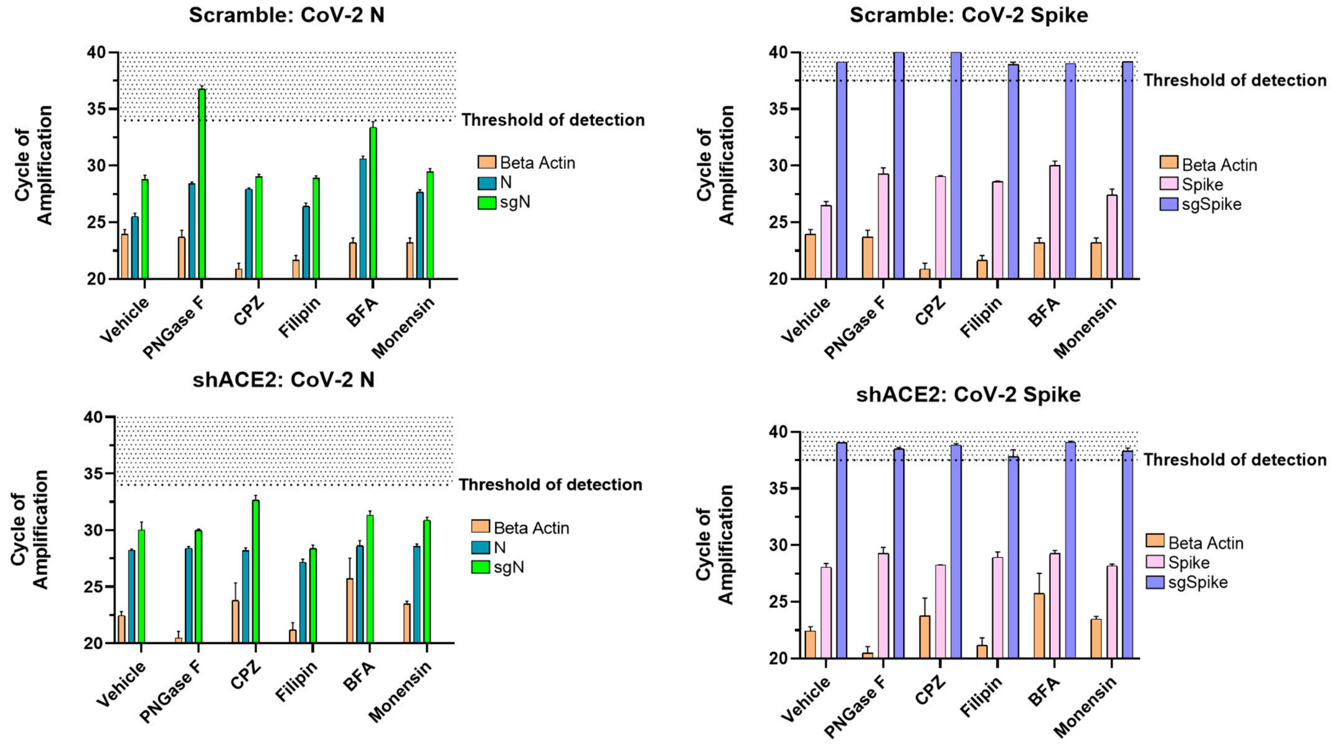

Supplementary Figure 3. Raw cycle of amplification values for genomic and subgenomic RNA from upper wells in lentivirus and drug experiments. The threshold of detection was established as 2 cycles below non-template control. Subgenomic N threshold = 34, and subgenomic spike threshold = 37.5. Subgenomic spike was not detected in any sample.
